# P300/CBP-associated factor (PCAF) inhibits the growth of hepatocellular carcinoma by promoting cell autophagy

**DOI:** 10.1038/cddis.2016.247

**Published:** 2016-10-06

**Authors:** Yu-Li Jia, Meng Xu, Chang-Wei Dou, Zhi-Kui Liu, Yu-Mo Xue, Bo-Wen Yao, Ling-Long Ding, Kang-Sheng Tu, Xin Zheng, Qing-Guang Liu

**Affiliations:** 1 Department of Hepatobiliary Surgery, The First Affiliated Hospital of Xi'an Jiaotong University, 277 Yanta West Road, Xi'an 710061, Shaanxi, PR China

## Abstract

Aberrant autophagic processes have been found to have fundamental roles in the pathogenesis of different kinds of tumors, including hepatocellular carcinoma (HCC). P300/CBP-associated factor (PCAF), a histone acetyltransferase (HAT), performs its function by acetylating both histone and non-histone proteins. Our previous studies showed that PCAF was downregulated in HCC tissues and its high expression was significantly associated with patient survival after surgery, serving as a prognostic marker. In this study we found that overexpression of PCAF induced autophagy of HCC cells and its knockdown depressed autophagy. As type II programmed cell death, autophagy induced by PCAF-elicited cell death in HCC cells. *In vivo* experiments confirmed that PCAF-induced autophagy inhibited tumor growth. Subsequent *in vitro* experiments showed that PCAF promoted autophagy by inhibiting Akt/mTOR signaling pathway. Our findings show that PCAF is a novel modulator of autophagy in HCC, and can serve as an attractive therapeutic strategy of HCC treatment.

Hepatocellular carcinoma (HCC) is the fifth most common malignancy and the third leading cause of cancer mortality worldwide. Because of lack of effective approaches to treatment, late diagnosis and impaired liver function, the prognosis of HCC patients is dismal, with a 5-year survival rate at ~15%.^1^ Surgical resection and liver transplantation are regarded as the main curative treatments for HCC. However, curative treatments can only be performed in patients with early-stage disease who have localized tumors. For patients at advanced stage, the efficacy of chemotherapies is unsatisfactory because of the drug resistance of HCC cells. Therefore, the critical first step toward novel effective therapeutic modalities is to gain a better understanding of the development and progression of HCC.

As a physiological process, autophagy regulates the routine turnover of proteins and intracellular organelles.^2^ Multiple cell signaling pathways have been confirmed to maintain or regulate autophagy, including Akt/mTOR signaling.^3^ The process of autophagy starts by the sequestering of cytosolic proteins or organelles into autophagosomes, which then fuse with lysosomes to form autolysosomes for the degradation of sequestered contents by lysosomal hydrolases.^4^ This biological process may contribute to cancer progression by promoting the survival of nutrient-starved cells. On the other hand, autophagy may benefit cancer progression by promoting cell death, which has recently attracted much attention for its prominent roles in cell survival and cell death, particularly in the pathogenesis and the treatment of cancer.^4, 5^

P300/CBP-associated factor (PCAF) is a member of the GNAT (GCN5-related *N*-acetyltransferase) acetyltransferase family, which was originally identified through the research into the oncogenic function of adenoviral E1A.^6^ As a well-known histone acetyltransferases (HAT), PCAF was found to repress cellular transformation by competing with E1A for binding to P300/CBP. Our previous studies confirmed that PCAF was frequently downregulated in HCC tissues compared with adjacent liver tissues. The downregulation of PCAF in tumor specimens was associated with TNM stage and tumor metastasis and also had negative correlations with survival rate after liver resection.^7^ Moreover, we found that PCAF increased histone H4 acetylation and inhibited AKT signaling.^8^ Recently, histone deacetylase inhibitors has been found to induce cell autophagy in HCC cells.^9^ So we hypothesized a connection between PACF and autophagy in HCC.

In this study, we showed that PCAF induced autophagy in HCC cells through inhibition of the Akt/mTOR pathway. Blocking the process of autophagy reduced PCAF-induced cytotoxicity, indicating that PCAF elicited cell death by autophagy. Our data have shown that PCAF is an attractive candidate for the treatment of HCC and that pharmacological targeting of autophagy provides promise for the management of cancer therapy.

## Results

### PCAF promotes cell autophagy in HCC cells

**Figure 1:**
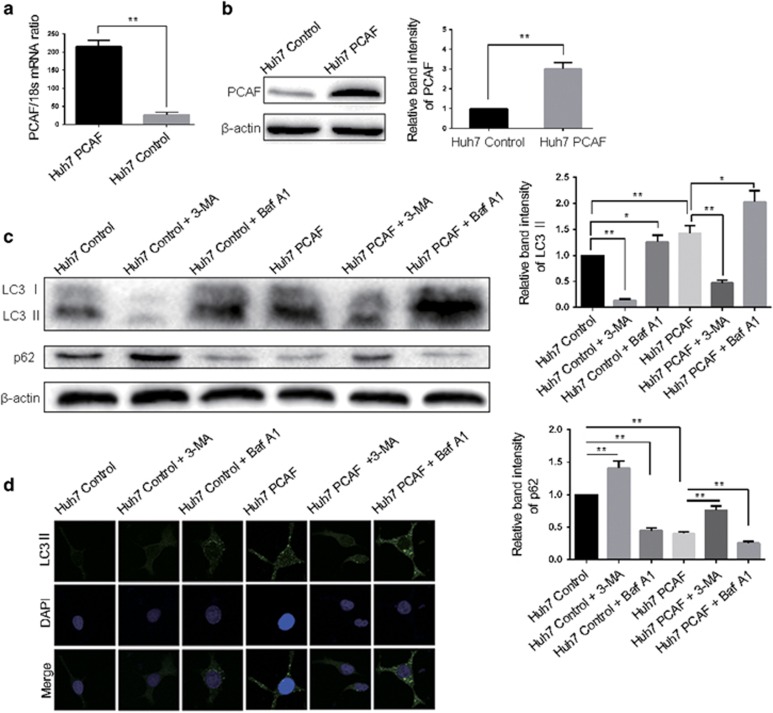
Overexpression of PCAF induced autophagy in HCC cells. PCAF expression was significantly increased by PCAF expressing plasmid in Huh7 cells at both the mRNA (**a**) and protein (**b**) level, compared with Huh7 Control cells. The results are summarized as relative band intensity of PCAF protein. The experiments were repeated at least three times. (**c**) Huh7 cells were transfected with plasmids expressing full-length PCAF mRNA or empty control plasmid, and Huh7 Control cells and Huh7 PCAF cells were treated with 5 mM 3-MA or 50 nM bafilomycin A1 for 24 h. Cell lysates were analyzed by immunoblotting for LC3 II and p62. In Huh7 cells, 3-MA decreased the expression of LC3 II and increased p62 expression, whereas the effect of bafilomycin A1 was opposite. The experiments were repeated at least three times. (**d**) The above-described cells were incubated with anti-LC3 antibody and were assessed by confocal microscopy. *P<0.05, **P<0.01

In our previous study we have already investigated the level of PCAF in HCC cell lines and selected the applicable cell models.^8^ Here, we selected Huh7 cells for PCAF overexpression experiment and Hep3B cells for PCAF knockdown experiment. To confirm whether PCAF regulates the autophagy of HCC cells, we established Huh7 clones that stably overexpressed PCAF by using the PCAF expressing plasmid. Figures 1a and b showed that the mRNA and protein expression of PCAF in Huh7 PCAF cells was significantly higher than in Huh7 Control cells. LC3, a subunit of microtubule-associated proteins 1A and 1B (termed MAP1 LC3), is a mammalian homolog of yeast autophagy-related protein 8.^10, 11^ In the process of autophagy, the transition of cytosolic LC3 I to autophagosomal membrane-binding protein LC3 II is essential for autophagosome formation.^12, 13, 14^ LC3 II levels are more reliable when measuring the degree of autophagy, as the expression of LC3 I can be transcriptionally regulated and LC3 I is less sensitive than LC3 II in western blotting assays. Because p62 can be degraded by autophagy, we further investigated the autophagic flux by the decrease of p62.^15^ Thereby, the effect of PCAF on autophagy would be measured by the expression of both LC3 II and p62. As Figure 1c showed, PCAF-induced LC3 II accumulation and p62 degradation were found in Huh7 PCAF cells compared with Huh7 Control cells. Both increased upstream autophagosome formation; blocked downstream autophagosome–lysosome fusion can result in the LC3 II accumulation.^16^ To make a distinction between these two possibilities, LC3 flux was measured in the presence of 3-MA or bafilomycin A1. 3-MA, a class III PI3K inhibitor blocking autophagosome formation, attenuated PCAF-induced LC3 II accumulation, whereas, PCAF could still cause the accumulation of LC3 II in the presence of bafilomycin A1, a vacuolar-type H^+^-ATPase inhibitor blocking autophagosome–lysosome fusion (Figure 1c). These suggested that the blockade of autophagic degradation was not the reason for the increase of LC3 II. The control effect of 3-MA alone and bafilomycin A1 alone were exhibited in Figure 1c. Moreover, an increase of LC3 II puncta that forms in the cytoplasm and represents autophagic vacuoles also provided evidence of autophagosome formation using immunofluorescence. Consistently, Figure 1d showed that PCAF induced an increase of LC3 II puncta in Huh7 cells, which could be attenuated by 3-MA and enhanced by bafilomycin A1.

**Figure 2:**
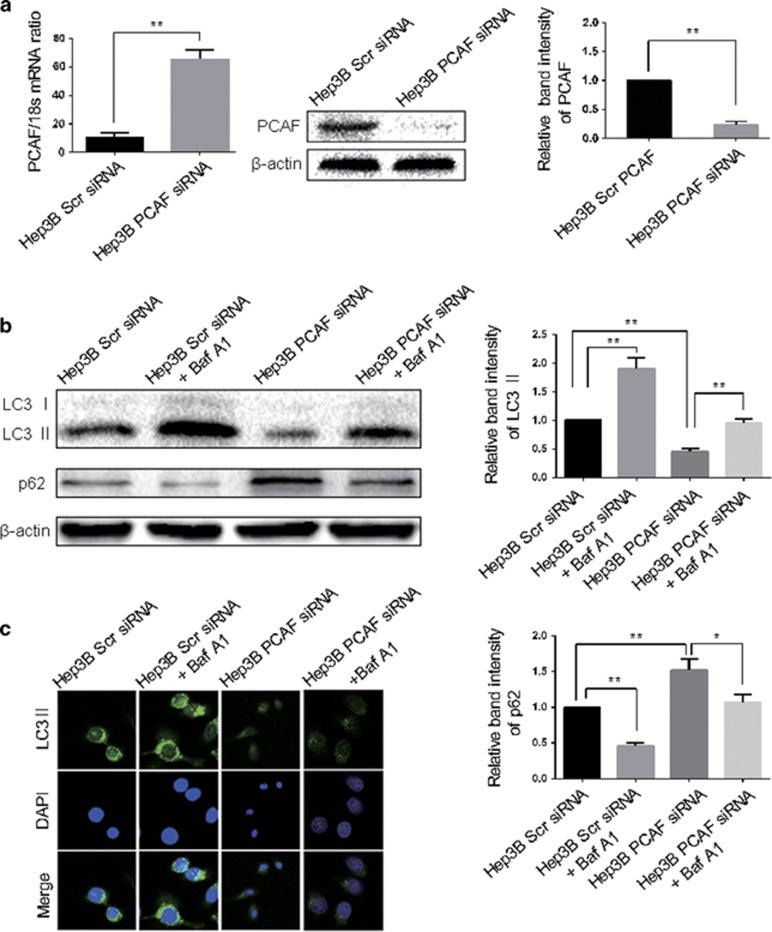
Knockdown of PCAF decreased autophagy in HCC cells. (**a**) PCAF expression was significantly decreased by PCAF siRNA in Hep3B cells at both the mRNA and protein level, compared with Hep3B Scr siRNA cells. The experiments were repeated at least three times. (**b**) In Hep3B Scr siRNA cells, Hep3B PCAF siRNA cells, Hep3B Scr siRNA cells treated with bafilomycin A1 and Hep3B PCAF siRNA cells treated with bafilomycin A1, the expression of LC3 II and p62 was assessed western immunoblotting. The experiments were repeated at least three times. (**c**) The LC3 II puncta in above cells were demonstrated by the confocal microscopy. *P<0.05, **P<0.01

To further determine the effect of PCAF on the cell autophagy of HCC cells, the expression of PCAF in Hep3B cells was silenced. Figure 2a showed that PCAF expression was decreased significantly by siRNAs transfection in Hep3B cells. Moreover, we observed attenuated accumulation of LC3 II, increased expression of p62 and decreased LC3 II puncta in Hep3B PCAF siRNA group, compared with Hep3B Scr siRNA group (Figures 2b and c). And the inhibition of autophagy induced by the knockdown of PCAF was reversed in the presence of bafilomycin A1 (Figures 2b and c). These results suggest that PCAF can induce autophagy in HCC cells.

### PCAF-induced autophagy leads to cell death and tumor growth supression *in vitro* and *in vivo*

**Figure 3:**
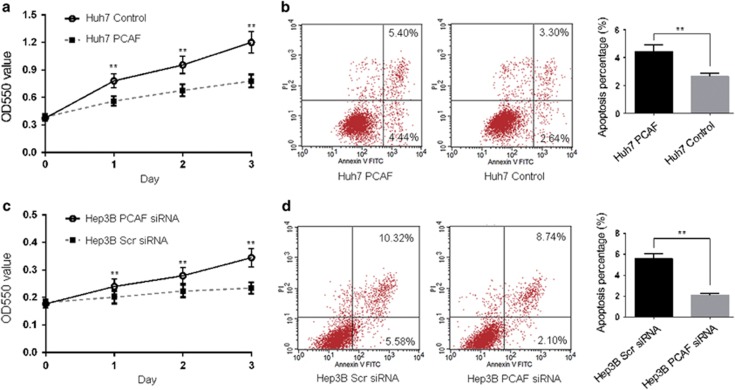
Forced expression of PCAF inhibited proliferation in Huh7 cells. (**a**) As MTT assays showed, overexpression of PCAF was found to significantly reduce the viability of Huh7 cells at 24 h, 48 h and 96 h. (**b**) Annexin V-FITC/PI labeling showed that forced expression of PCAF resulted in an increase in early apoptosis of Huh7 cells. The percentages of cells in the right lower quadrant were calculated as the percentage of apoptosis in the quantitative graph. The experiments were repeated at least three times. The viability of Hep3B PCAF siRNA cells and Hep3B Scr siRNA cells was also detected by MTT assay (**c**) and flow cytometry (**d**). The experiments were repeated at least three times. **P<0.01

Autophagy protects against malignancies by inducing cell death (type II programmed cell death) or contributes to malignancies by promoting the survival of nutrient-starved cells.^4^ To determine the effect of PCAF-induced autophagy on the survival of HCC cells, we performed MTT assay and flow cytometry apoptosis assays. As shown in Figure 3a, cell viability was notably reduced in Huh7 PCAF cells compared with Huh7 Control cells. We observed that 4.44% of the cells underwent early apoptosis in Huh7 PCAF cells, whereas only 2.64% of Huh7 Control cells were apoptotic (Figure 3b). On the other hand, knockdown of PCAF was found to promote proliferation of Hep3B cells (Figure 3c). Figure 3d demonstrates that early apoptosis of Hep3B PCAF siRNA cells was decreased to 2.4%, whereas the rate of apoptosis was 5.58% in Hep3B Scr siRNA cells. These results suggested that PCAF-induced autophagy accounted for cell death.

**Figure 4:**
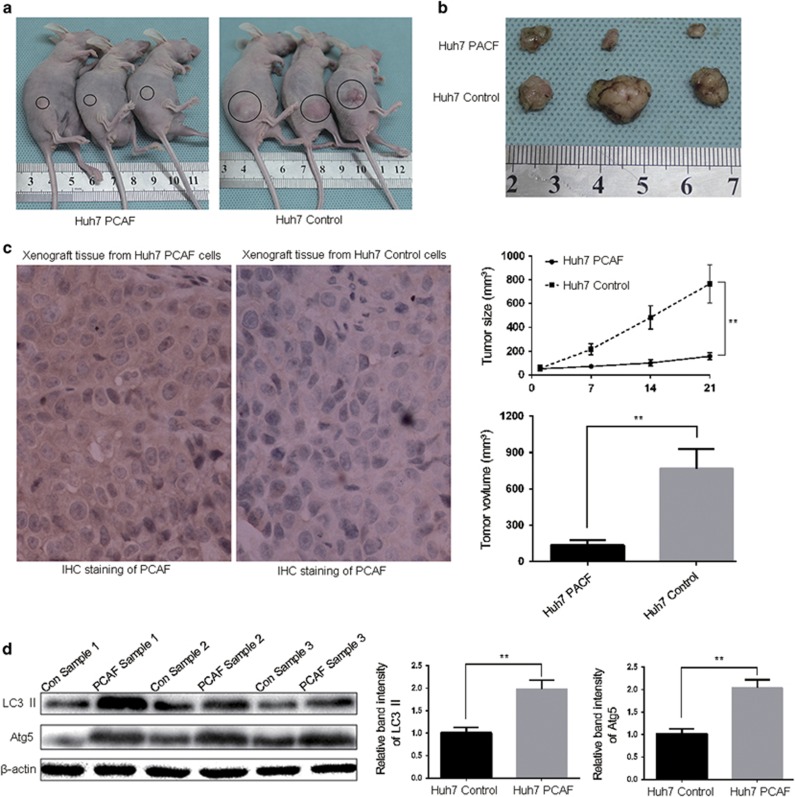
HCC xenograft growth was repressed by PCAF *in vivo*. Representative tumor-bearing mice (**a**) and explanted tumors (**b**) are shown. The tumor growth curves, tumor size and volume are plotted in (**b**), respectively. (**c**) Tumor nodules were subjected to immunohistochemical staining for PCAF. (**d**) Western blot showing LC3 II and Atg5 levels from cellular lysates isolated from xenografts from Huh7 PCAF cells and Huh7 Control cells. Con Sample: tissue sample from xenografts from Huh7 Control cells. PCAF Sample: tissue sample from xenografts from Huh7 PCAF cells. The experiments were repeated at least three times. **P<0.01

**Figure 5:**
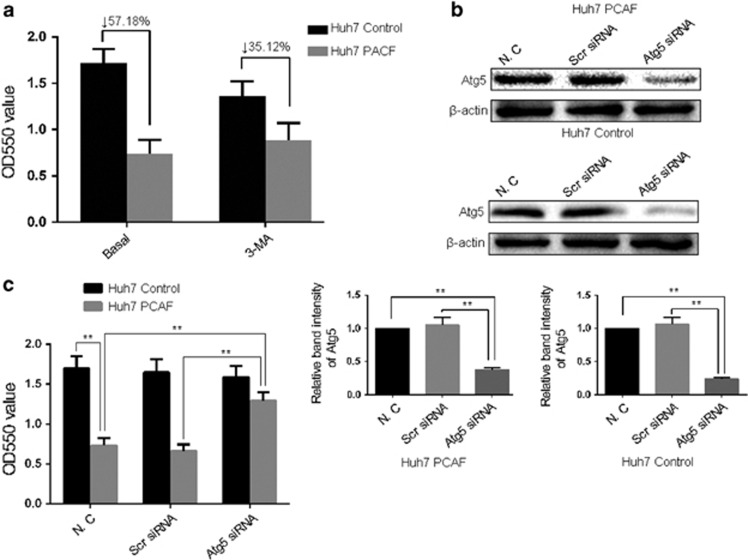
PCAF-induced cell death was reversed by the inhibition of autophagy. (**a**) Huh7 PCAF cells and Huh7 Control cells were treated in combination with 5 mM 3-MA for 3d before MTT assay performed. The experiments were repeated at least three times. (**b**) Atg5 expression was significantly decreased by Atg5 siRNA in Huh7 PCAF cells and Huh7 Control cells at the protein level. (**c**) MTT assays showed that knockdown of Atg5 was found to significantly increase the cell viability of Huh7 PCAF cells. **P<0.01

To further confirm that PCAF induced autophagy *in vivo*, Huh7 PCAF cells and Huh7 Control cells were used to develop a subcutaneous tumor model. As shown in Figures 4a and b, the size and volume of xenografts derived from Huh7 PCAF cells was significantly smaller than that those from Huh7 Control cells at the 21 day after HCC cells injection. Tumor growth curves of xenografts from Huh7 PCAF cells were significantly slower than xenografts from Huh7 Control cells (Figure 5b). As shown in Figure 4c, the expression of PCAF protein was strongly positive in the xenografts from Huh7 PCAF cells, whereas negative PCAF staining was found in the xenografts from Huh7 Control cells. Moreover, increased LC3 II and Atg5 protein levels confirmed the appearance of autophagy in the xenografts from Huh7 PCAF cells (Figure 4d). These results showed that PCAF-induced autophagy supressed tumor growth *in vivo*.

### Inhibition of autophagy reversed PCAF-induced cytotoxicity

To clarify the role of autophagy in PCAF-induced cytotoxicity, we first treated Huh7 PCAF cells with 3-MA and conducted the MTT assay. PCAF reduced the cell viability by 56.59%, which was rescued by 3-MA (Figure 5a).Then we created Atg5 knockout cells model to further confirm that the inhibition of autophagy can significantly decrease PCAF-induced cell cytotoxicity. Figure 5b showed that Atg5 was obviously decreased by siRNAs transfection in Huh7 PCAF cells and Huh7 Control cells. Then with knockdown of the essential autophagy genes Atg5, we determined the cell viability by MTT assay (Figure 5c). These data demonstrated that the inhibition of autophagy can reduce PCAF-induced cytotoxicity of HCC cells.

### Akt/mTOR signaling has an important role in PCAF-induced autophagy of HCC cells

**Figure 6:**
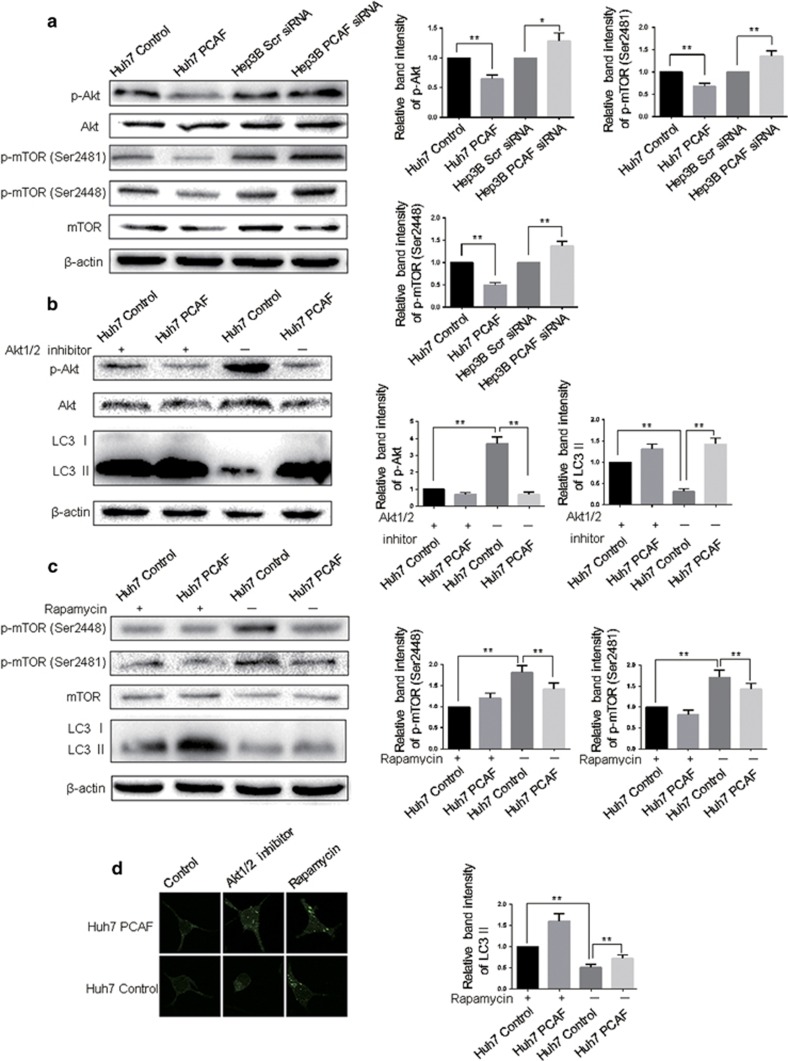
PCAF inhibited Akt/mTOR signaling. (**a**) Huh7 PCAF cells, Huh7 Control cells, Hep3B PCAF siRNA cells and Hep3B Scr siRNA cells were analyzed for Akt/mTOR activity by western blotting assay. The experiments were repeated at least three times. Huh7 PCAF cells and Huh7 Control cells in presence or absence of 10 *μ*M Akt1/2 inhibitor (**b**) and 10 nM rapamycin (**c**) were also analyzed for Akt/mTOR activity by western blotting assay. The experiments were repeated at least three times. (**d**) LC3 II puncta in the above-described cell lines were imaged by the confocal microscopy. *P<0.05, **P<0.01

Inhibition of Akt/mTOR signaling is a well-recognized pathway to induce autophagy.^2, 17^ To investigate the molecular mechanisms of PCAF-induced autophagy, Akt and mTOR kinase activity, as measured by their phosphorylation, was first examined. PCAF expression inhibited the phosphorylation of Akt and mTOR kinase, whereas knockdown of PCAF stimulated Akt and mTOR kinase activity (Figure 6a). These findings suggested that PCAF could elicite autophagy in HCC cells via decreasing the activation of the Akt/mTOR pathway. To ensure the regulatory role of Akt/mTOR pathway in PCAF-induced autophagy, Huh7 PCAF cells and Huh7 Control cells were treated with Akt1/2 inhibitor and rapamycin. As shown in Figure 6b, inhibition of Akt1/2 increased autophagic signaling, which was indicated by additionally enhanced LC3 II level in Huh7 PCAF cells. Similarly, mTOR inhibition by rapamycin resulted in decreased phosphorylation of target protein and enhanced the autophagic effect of PCAF (Figure 6c). Furthermore, inhibition of Akt/mTOR signaling pathway or overexpression of PCAF could increase the percentage of LC3 II puncta as a marker of autophagy induction (Figure 6d). Taken together, the above results suggested that PCAF suppressed Akt/mTOR pathway activity to induce autophagy.

### PCAF induced autophagy and apoptosis in a parallel manner

**Figure 7:**
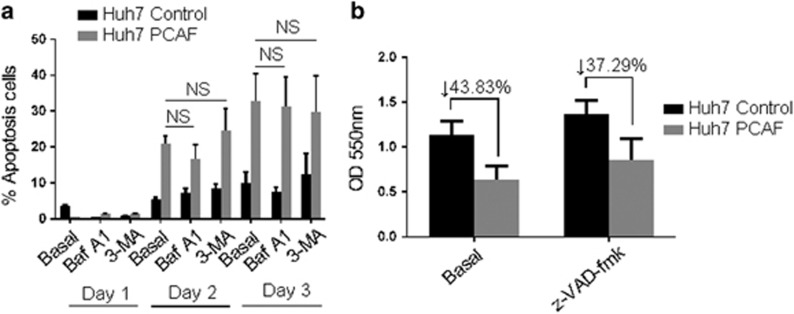
Effect of 3-MA, bafilomycin A1 and z-VAD-fmk on PCAF-induced apoptosis. (**a**) Huh7 PCAF cells and Huh7 Control cells in presence or absence of 5 mM 3-MA or 50 nM bafilomycin A1 (Baf A1) for 1–3 days, and then the Annexin V-FITC/PI double-staining analysis was performed. Cells in early apoptotic and necrotic/late apoptotic phases were quantified as apoptotic cells. The experiments were repeated at least three times. (**b**) Huh7 PCAF cells and Huh7 Control cells in presence or absence of 100 nM z-VAD-fmk for 2 days. Cytotoxicity was assessed by MTT assay. The experiments were repeated at least three times

Previous studies demonstrated that autophagy and apoptosis may be prompted in an independent or mutually exclusive manner, and the cell fate is decided by these two phenomena cooperatively.^18^ Therefore, to further investigate whether PCAF also induced apoptosis we conducted Annexin V-FITC/PI double staining. Apoptosis was observed in Huh7 cells after transfection with PCAF plasmid for 2 and 3 days, but PCAF-induced apoptosis was not affected by 3-MA or bafilomycin A1 treatment (Figure 7a). Moreover, the pan-caspase inhibitor z-VAD-fmk reversed PCAF-induced cytotoxicity (Figure 7b). These results indicated that PCAF simultaneously induced autophagy and apoptosis, and they occurred independently.

## Discussion

Accumulating evidences indicate that autophagy deficiency is involved in malignant clinicopathological characteristics and poor prognosis of HCC; and that autophagy can contribute to suppression of tumor development and progression.^19, 20^ Thus, reactivation of autophagy in tumor cells could be an effective therapy for HCC. In this study our results demonstrated that PCAF induced autophagy in HCC and that combination with 3-MA or knockdown of Atg5 reduced the cytotoxicity of PCAF, suggesting that PCAF induced cell death by autophagy.

To monitor the level of autophagy, it is better to measure the overall levels of LC3 II and p62 and the formation of LC3-specific puncta according to numerous studies of autophagy detection.^12, 13, 14, 21, 22^ Treatment with 3-MA or Baf A1 were necessary to identify different processes of autophagy. Suitable alteration of autophagy, such as inhibition of cytoprotective autophagy or promotion of cell death, might enhance the cytotoxicity of anti-cancer therapy. We found that PCAF-induced autophagy led to cell death in HCC cells and the inhibition of autophagy via Atg5 knockdown suppressed PCAF-induced cytotoxicity. Moreover, in the Huh7 xenograft model we found that xenografts with high PCAF expression grew significantly slower than those with low PCAF expression, and the levels of LC3 II and Atg5 in the xenografts with high PCAF expression were increased. Therefore, combination with autophagic inducers may enhance the efficacy of PCAF on cancer therapy.

The Akt/mTOR pathway has been involved in regulating cell apoptosis and autophagy.^23, 24^ Phosphorylation by upstream kinases is essential for complete activation of Akt and activated Akt in turn relieves mTOR inhibition by phosphorylating its negative regulators.^25, 26^ The function of mTOR is also regulated by phosphorylation at Ser2448 and at Ser2481.^27, 28^ In this study, we observed that downregulation of AKT and mTOR phosphorylation occurred in the presence of PCAF and additional increases in LC3 II levels upon inhibition of AKT and mTOR kinase activity. These results imply that PCAF inhibits Akt/mTOR pathway to induce autophagy. Further study is needed to clarify clear mechanism about PCAF-induced autophagy and the Akt/mTOR signaling pathway.

The HAT PCAF has been shown to be involved in the modulation of differentiation, angiogenesis, cell cycle progression, gluconeogenesis and carcinogenesis.^8, 16, 29, 30^ Our previous study indicated that PCAF protein was bound with histone H4 protein, acetylated histone H4 directly and inhibited AKT signaling in Huh7 cells. Yuan-Ling Liu and others found that two potent HDAC inhibitors induced autophagy in HCC cells through downregulation of Akt/mTOR signaling.^9^ In addition, increasing numbers of studies revealed that acetyltransferase enzymes also acetylate non-histone proteins, including p53, importin-α adaptor, E1A viral oncoprotein, FOXP3 and Gli1.^31, 32, 33, 34, 35, 36^ So PCAF may regulate autophagy through acetylating histone protein or other non-histone protein related with autophagy including Key Atg Genes. Further studies are necessary to elucidate deeper mechanism about how PCAF induced autophagy, including whether PCAF-mediated autophagy was the consequence of a transcriptional modulation.

In summary, this study demonstrates that PCAF induces autophagy (in addition to apoptosis) to mediate cancer cell death. Furthermore, PCAF was found to downregulate Akt/mTOR signaling pathway to activate autophagy. We also found that PCAF enhances cytotoxity and suppresses tumor growth. Taken together, our results reiterate that PCAF may be a potential clinical candidate for the treatment of HCC and we also provide first evidence of autophagy as an underlying mechanism.

## Materials and Methods

### Cell culture and reagents

Huh7 and Hep3B cells were purchased from the Institute of Biochemistry and Cell Biology, Chinese Academy of Sciences, Shanghai, China. Cells were maintained in Dulbecco's modified Eagle's medium (Gibco, Grand Island, NY, USA) supplemented with 10% fetal bovine serum (Gibco), 100 units/ml penicillin and 100 mg/ml streptomycin in a humidified incubator at 37 °C with 5% CO_2_. Bafilomycin A1 (Sigma, St-Louis, MO, USA) and 3-MA (Sigma) were used at 50 nM and 5 mM, respectively. Akt1/2 kinase inhibitor was also purchased from Sigma. Rapamycin was procured from Millipore Corporation (Billerica, MA, USA). The following primary antibodies from Cell Signaling Technology (Danvers, MA, USA) were used: PCAF (#3378), Akt (#2920), phospho-Akt (#4060), mTOR (#2972), phospho-mTOR (Ser2448) (#2971), phospho-mTOR (Ser2481) (#2974), LC3-A/B (#4108), β-actin (#12262) and Atg5 (#8540).

### Cell transfection and RNA interference

PCAF-expressing plasmid and empty plasmid pCMV6-Entry were purchased from Origene Technologies Inc (Rockville, MD, USA). Huh7 cells were transfected with plasmids using Lipofectamine 2000 according to the manufacturer's instructions (Invitrogen, Carlsbad, CA, USA). Stable transfection was achieved as described previously.^8^ The siRNAs used in this investigation were purchased from Santa Cruz Biotechnology (Santa Cruz, CA, USA): *PCAF* siRNA (Catalog No. sc-36198), Atg5 siRNA (Catalog No. sc-41446) and scrambled siRNA (Catalog No. sc-37007). Transfection was performed as described previously.^8^

### Quantitative real-time reverse transcription polymerase chain reaction (qRT-PCR)

Total RNA was extracted from HCC cell lines with TRIzol reagent (Invitrogen) according to the manufacturer's protocol. cDNA synthesis and qRT-PCR analysis were done as described.^37^ The ABI TaqMan probes (Applied Biosystems, Carlsbad, CA, USA) targeting *PCAF* (Hs00187332_m1) and 18 s rRNA (Hs99999901_s1) were used here. The relative expression of *PCAF* was shown as fold difference relative to 18 s rRNA. There were six replicates in each measurement and the assessment was repeated three times.

### Western blot analysis

Cells and xenograft tumor tissues were lysed on ice in RIPA lysis buffer (50 mM Tris pH 7.5, 150 mM NaCl, 1% TritonX-100, 5 mM ethylenediaminetetraacetic acid). Cell lysates were then centrifuged at 12 000 × *g* for 15 min at 4 °C. Supernatant was collected and protein concentration was measured using a BCA Kit (Pierce, IL, USA). Equal amounts of protein (50 *μ*g) were separated by SDS-PAGE and transferred onto PVDF membranes. Membranes were incubated with the corresponding primary antibody at 4 °C overnight. Then, the membranes were washed and incubated with horseradish peroxidase-conjugated goat anti-mouse or anti-rabbit secondary antibodies (1:5000–1:10000, BioRad, Hercules, CA, USA) for 2 h at room temperature. Specific protein bands were measured with an enhanced chemiluminescence reagent (Millipore Corporation) and visualized by a chemiluminescence detector (Bio-Rad Laboratories, Inc., Berkeley, CA, USA) that can also assess the relative intensity of the bands.

### Immunofluorescence

Huh7 or Hep3B cells, grown on coverslips, were then rapidly washed with PBS and fixed with ice-cold 100% methanol for 15 min at −20 °C, then blocked in 1% BSA for 1 h. Immunolabeling with the anti-LC3-A/B antibody was performed by incubation at −4 °C overnight. Secondary labeling was performed with goat anti-rabbit FITC (green) IgG antibody (ZSGB-BIoInc., Beijing, China) at 1:100 dilution for 60 min. Imaging was with a Leica TCS SP5 laser-scanning confocal microscope with LAS-AF imaging software, using a × 63 oil objective.

### Immunohistochemical staining

Tissues were fixed in formalin and embedded in paraffin before sectioning. In brief, the sections (4 *μ*m) were deparaffinized in xylene and rehydrated by stepwise washes in decreasing ethanol concentrations, quenched for endogenous peroxidase in 3% hydrogen peroxide, followed by boiling in retrieval solution to expose antigens and then slides were incubated with the primary antibody against PCAF overnight at 4°C. The reaction was visualized with diaminobenzidine and counterstained with hematoxylin, which resulted in a brown-colored stain at the site. All slides were stained with hematoxylin and eosin for histologic analysis and observed by two pathologists independently to evaluate the results. The overall intensity and percentage were evaluated at 10 independent fields (× 400).

### *In vivo* experiments

Four-to-six week-old female BALB/c nude mice (Centre of Laboratory Animals, The Medical College of Xi'an Jiaotong University, Xi'an, China) were used to establish the nude mouse xenograft model, and were kept in laminar-flow cabinets under specific pathogen-free conditions, and handled according to the recommendations of the National Institutes of Health guidelines for care and use of laboratory animals. For the tumor challenge, 2 × 10^6^ Huh7 PCAF cells or Huh7 Control cells suspended in 150 *μ*l of Matrigel were inoculated subcutaneously into the flanks of nude mice, and tumor growth was monitored each week. There were three animals in each group. The mice were killed on day 21 after implantation. Tumor tissues were prepared for western blot analysis and immunostaining.

### Cell viability assays

MTT (3-(4, 5-dimethylthiazol-2-yl)-2, 5-diphenyltetrazolium bromide; Roche, USA) assays were used to measure the cell viability. In total, 2 × 10^3^ HCC cells were plated in at least triplicate in 96-well plates and treated with *PCAF-*expressing plasmid or *PCAF* siRNA. After 24 h, 48 h and 72 h incubation, 0.5 mg/ml of MTT was added to each well for an additional 4 h period. The blue MTT formazan precipitate was then dissolved in 100 *μ*l of DMSO. The absorbance at 550 nm was measured on a multiwell plate reader.

### Flow cytometry

HCC cells were grown to 50% confluency, transfected with *PCAF*-expressing plasmid for indicated time intervals. The cells were trypsinized, washed in PBS and centrifuged at 200 × *g* for 5 min. Then, the pellet was resuspended in 100 *μ*l binding buffer, and 2 *μ*l Annexin V-FITC and 2 *μ*l propidium iodide (PI) was added. After 15 min incubation at room temperature, FITC and PI fluorescences were detected by FACScalibur flow cytometer (Becton Dickinson, San Diego, CA, USA) and subsequently analyzed by CellQuest software.

### Ethics statement

The protocols used in this study were reviewed and approved by Xi'an Jiaotong University Ethics Committee according to the Helsinki Declaration of 1975.

### Statistical analysis

All data are presented as the Mean±S.E.M. The SPSS statistical package for Windows Version 13 (SPSS, Chicago, IL, USA) was used for the two-tailed Student's *t*-test to evaluate statistical significance using GraphPad Prism 5 software (GraphPad Software, Inc, San Diego, CA, USA). *P*-values<0.05 was considered to be statistically significant. Means and S.D.s of samples (performed in at least three times) were calculated from the numerical data generated in this study.

## Abbreviations

PCAF: P300/CBP-associated factor
HCC: hepatocellular carcinoma
HATs: histone acetyltransferases
Atg: autophagy-related protein
LC3: microtubule-associated protein 1 light chain 3
